# The Gut-Brain Axis in Neurodegeneration: Mechanistic Links Between Dysbiosis and Neuropathology

**DOI:** 10.22034/ijp.2026.2081887.3613

**Published:** 2026-08-01

**Authors:** Maryam Azimzadeh, Shima Ababzadeh, Ali Ganji Kahaki, Seyyed Mohammad Hoseine Babaei, Reihaneh Seyedebrahimi, Mohsen Eslami Farsani

**Affiliations:** 1 Department of Medical Laboratory Sciences, Khomein University of Medical Sciences, Khomein, Iran; 2 Cellular and Molecular Research Center, Qom University of Medical Sciences, Qom, Iran; 3 Tissue Engineering and Applied Cell Sciences Department, School of Medicine, Qom University of Medical Sciences, Qom, Iran; 4 Student Research Committee, Qom University of Medical Sciences, Qom, Iran; 5 Anatomy Department, Faculty of Medicine, Qom University of Medical Sciences, Qom, Iran

**Keywords:** Gastrointestinal Microbiome, Dysbiosis, Brain, Neurodegenerative Diseases, Inflammation, Gut-Brain Axis

## Abstract

**Background & Objective:**

The gut-brain axis is essentially a two-way communication system that physically connects the brain and the intestinal tract. The connection is mediated through a series of pathways, including neural, endocrine, and immune pathways. Gut dysbiosis, which is explained as an imbalance in the microbial community, has been linked to the causation of various neurodegenerative disorders such as Alzheimer’s disease, Parkinson’s disease, and multiple sclerosis. However, the pathological mechanisms in the brain are only partially known. The present review outlines the process of gut dysbiosis and neurodegeneration, detailing the roles of protein aggregation, neuroinflammation, barrier disruption, and neuroglial dysfunction. Then, extending the comparison to a range of neurodegenerative diseases, we discuss the possibility of common pathway therapeutics and actual microbiome-based treatment options planning from the standpoint of microbiome-directed interventions.

**Content/Findings:**

Gut dysbiosis triggers a definable cascade, starting with the disruption of the intestinal barrier and increased permeability (leaky gut), which allows bacterial products (lipopolysaccharides, bacterial amyloids) and pro-inflammatory cytokines to enter systemic circulation. Such peripheral changes weaken the blood-brain barrier and thus allow these factors to access the CNS, where they lead to neuroglial dysfunction (microglial priming, astrocytic reactivity, and oligodendrocyte injury) by disruption of glial homeostasis. CNS glial cell malfunction leads to the development of proteinopathies characteristic of each disease: amyloid and tau hyperphosphorylation in Alzheimer’s disease through BACE1 upregulation and kinase activation; synuclein in Parkinson’s disease via molecular mimicry, oxidative stress, and impaired clearance; and demyelination in multiple sclerosis through oligodendrocyte apoptosis. Oral bacteria such as Porphyromonas gingivalis aggravate this inflammatory loop through the direct invasion of the CNS and proteolytic cleavage of amyloid and tau. The vagus nerve is yet another pathway through which gut-derived inflammatory signals and pathological synuclein can be transmitted to the brain.

**Conclusion:**

The gut microbiome is more than just a correlate of neurodegeneration; it actively promotes neurodegenerative diseases through pathways that can be mechanistically defined. Microbiome-targeted interventions such as dietary changes, precision probiotics, fecal microbiota transplantation, and anti-inflammatory agents offer a measure of hope for changing these pathological processes. Future studies need to be directed at determining the time sequence of cause and effect, finding dependable microbiota-based biomarkers, and formulating tailored strategies that can account for individual microbial composition variability, genetic susceptibility, and environmental exposures. A deeper understanding of the gut-brain axis from this mechanistic perspective could eventually lead to the prevention or postponement of neurodegeneration.

## 1. Introduction

The gut-brain axis represents one of the most fascinating and rapidly evolving frontiers in modern medicine. This intricate bidirectional communication network connects the gastrointestinal tract, its resident microbial communities, and the central nervous system (CNS) through neural, endocrine, and immune pathways(1). Besides being a main feature in neurological disorders, this axis fundamentally determines diverse physiological functions such as metabolism, immune homeostasis, and the body's overall health(2). Emerging evidence suggests that disturbances in this axis, particularly gut dysbiosis, play a significant role in the development and progression of neurodegenerative diseases such as Alzheimer’s disease and Parkinson’s disease. Understanding these connections provides critical insights into potential preventive and therapeutic strategies(3).

At the core of the gut-brain axis lies the gut microbiome, a diverse ecosystem of bacteria, viruses, fungi, and other microorganisms that reside in the human digestive tract. These microbes are not passive inhabitants but active participants in host physiology, influencing digestion, immunity, and even brain function. Under normal conditions, the microbiome functions in such a way as to ferment dietary fiber, synthesize vitamins, and train the immune system(4). The microbiome begins to establish itself at birth, shaped by factors such as delivery mode, diet, antibiotics, and environmental exposures. Over time, disruptions in microbial balance, known as dysbiosis, can lead to systemic inflammation, metabolic dysfunction, and impaired neurological health(5). Notably, gut dysbiosis is linked to neurodegeneration via microbial-induced inflammation, as a healthy gut prevents harmful substances from entering the bloodstream. However, dysbiosis can compromise this barrier, leading to increased intestinal permeability, often referred to as leaky gut(6). Importantly, the impact of oral and gut pathogens on neurodegeneration is a convergence of pathways that also operate in other inflammation-associated disorders(7). When the gut lining becomes permeable, bacterial endotoxins, such as lipopolysaccharides, can translocate into the systemic circulation, triggering an immune response. This systemic inflammation can then propagate to the brain, where it may contribute to neuroinflammation, a hallmark of neurodegenerative diseases(8,9). Chronic activation of brain immune cells, particularly microglia, can lead to neuronal damage and accelerate cognitive decline(10). Another critical aspect of the gut-brain axis involves microbial metabolites, particularly short-chain fatty acids. Short-chain fatty acids produced by gut bacteria from dietary fiber play a crucial role in maintaining gut barrier health, regulating the immune response, and supporting brain function. Butyrate, in particular, supports the blood-brain barrier (BBB), reduces neuroinflammation, and promotes brain-derived neurotrophic factor, which is essential for neuron survival and adaptability(11). Conversely, a deficiency in these protective metabolites, often seen in dysbiosis, may leave the brain more vulnerable to degenerative processes(12). The gut microbiome also interacts with the CNS through the vagus nerve, a major neural pathway that transmits signals between the gut and the brain. This nerve acts as a direct communication line, allowing gut microbes to influence mood, cognition, and stress responses(13). Furthermore, gut microbes generate neurotransmitters, including serotonin, dopamine, and gamma-aminobutyric acid (GABA), that are vital for brain function. Dysbiosis affects the synthesis of these neuroactive substances, possibly leading to neurological and psychiatric conditions(14). In addition, growing evidence suggests the involvement of certain pathogenic bacteria in neurodegenerative diseases. An example is the bacterium Porphyromonas gingivalis, which causes periodontal disease and has been found in the brains of patients with Alzheimer’s disease and is associated with the accumulation of toxic proteins such as β-amyloid. Similar changes in gut bacterial composition have been demonstrated in patients with Parkinson's disease (PD), and there is some evidence that gut dysfunction may occur several years before the onset of motor symptoms. These findings suggest that the gut may function as an early biomarker and even a regulatory target for neurodegenerative diseases(14,15).

Given these connections, interventions aimed at restoring gut microbial balance hold promise for mitigating neurodegeneration. Dietary modifications, such as increasing the intake of fiber-rich and fermented foods, can promote the growth of beneficial bacteria and enhance the production of protective metabolites, including short-chain fatty acids. Probiotics and prebiotics may also help reestablish a healthy microbiome, while fecal microbiota transplantation is being explored as a potential treatment for severe dysbiosis. Additionally, lifestyle factors such as stress management and physical activity can positively influence gut-brain communication(16). Accordingly, the gut-brain axis showcases the connection between gut health and brain function, where imbalances in gut microbiota and gut barrier integrity are associated with neuroinflammation and neurodegenerative diseases(17).

## 1.1. Aim of the Review

The rapidly growing domain of microbiome studies has clearly demonstrated a connection between gut dysbiosis and the clinical manifestations of neurodegenerative diseases. Despite this, it is still a major issue that no one has systematically linked specific disruptions within the gut ecosystem to the precise neuropathological cascades that define these disorders. Hence, the main focus of the present review is to discuss an updated mechanistic understanding that reveals how gut microbiome dysbiosis might be a driving factor in the neuropathology of neurodegeneration. Therefore, through the integration of evidence from these various fronts, this review aims to critically examine the mechanistic links between gut dysbiosis and neurodegeneration, evaluate the role of specific pathogens and metabolites, and discuss emerging therapeutic strategies targeting the gut-brain axis.

## 1.2. Literature Search Strategy

To gather a comprehensive overview of the present evidence, a formal literature search was undertaken. The major electronic databases searched included PubMed/MEDLINE, Scopus, and Web of Science. The search was limited to articles published from 2013 to 2026. A mixture of MeSH terms and keywords was used in the search strategy, such as: (“Gastrointestinal Microbiome” OR “Dysbiosis”) AND (“Neurodegenerative Diseases” OR “Alzheimer Disease” OR “Parkinson Disease” OR “Multiple Sclerosis”) AND (“Neuroinflammation” OR “Blood-Brain Barrier” OR “Microglia” OR “Protein Aggregation, Pathological”).

The initial screening consisted of a title and abstract review by two independent reviewers. Articles that appeared to be relevant were sought in full text and checked against the inclusion criteria set in advance:(1) original research papers, meta-analyses, or well-argued reviews;(2) publications that examined mechanistic pathways between gut flora and neurodegenerative disease in both animal and human studies; and(3) papers presented in the English language. The exclusion criteria were:[1] studies that addressed only psychiatric disorders without neurodegenerative aspects;[2] articles that did not offer a mechanistic explanation (for example, purely descriptive population studies without pathological correlates); and[3] conference abstracts, dissertations, and commentaries that had not undergone peer review.

## 2. Microbial Influence on Neuroinflammation: Gut Pathogens Focus

## 2.1. Gut Pathogens and Neurodegeneration

The gut microbiome is indispensable in modulating the key neuropathological aspects of Alzheimer's disease (AD) by multiple tightly interlinked mechanisms. Short-chain fatty acids (SCFAs), especially butyrate derived from commensal bacteria, serve as epigenetic mediators that help control amyloid pathology. Butyrate is a histone deacetylase inhibitor, and its lack in dysbiosis results in upregulation of BACE1, thus increasing amyloid production. On the other hand, butyrate treatment promotes the expression of amyloid-degrading enzymes and lowers the amyloid load, as evidenced by studies in germ-free mouse models(18).

The microbiome is equally essential in determining microglial behavior, as shown by the administration of specific probiotic strains that induce an anti-inflammatory, phagocytic phenotype beneficial for amyloid removal through the upregulation of receptors such as TREM2. On the other hand, the increase in circulating lipopolysaccharide due to dysbiosis brings microglia into a prolonged pro-inflammatory state with diminished phagocytic ability, thereby speeding up amyloid deposition and tau phosphorylation(19). The oral bacterium Porphyromonas gingivalis is a significant contributor to AD pathogenesis via its gingipain proteases, which cut tau into neurotoxic fragments and raise amyloid levels. These proteases have been detected in more than 90% of postmortem AD brains, and their blockade alleviates the pathology in animal models, thereby illustrating the existence of an oral-brain axis in disease progression(5).

Systemic inflammation due to gut dysbiosis is one of the mechanisms that promotes tau pathology by both activating kinases that hyperphosphorylate tau and changing bile acid metabolism, resulting in loss of inhibition of these pathways. Furthermore, such an inflammatory environment may facilitate the spread of pathological tau from one neuron to another through exosomes, mediated by these mechanisms(20). Moreover, some bacterial species in the gut are capable of synthesizing functional amyloids that, through molecular mimicry, can cross-seed amyloid aggregation, hence providing a direct means by which microbial products can trigger or potentiate cerebral amyloidosis(21). In sum, these studies reveal that the gut microbiome is an active player in the regulation of the generation, posttranslational modification, and removal of AD-associated proteins, thus providing a plethora of potential therapeutic targets for the treatment of the underlying neuropathology.

## 2.2. Gut Pathogens and Their Mechanisms

The slow, repeated accumulation of wrongly folded synuclein proteins into Lewy bodies and Lewy neurites is the major neuropathological feature of Parkinson's disease (PD). Increasing evidence now indicates a “gut-first” scenario, where the pathology originates outside the brain, more specifically in the neurons of the enteric nervous system. From this perspective, an imbalance of the gut microbiota is considered to be the key initiator, being responsible for establishing a local microenvironment that favors the very first misfolding of synuclein occurring in the gut wall(22). It is further assumed that this prion-like propagation of the pathology continues along the vagus nerve in a retrograde direction all the way to the dorsal motor nucleus, from where it moves up through the more vulnerable brainstem regions toward the substantia nigra and cortex, thus following the traditional Braak staging pattern. This gut-to-brain route is corroborated by the clinical finding that gastrointestinal dysfunctions such as chronic constipation can precede motor symptoms by several decades, and also by the presence of phosphorylated α-synuclein detected in enteric neurons and the vagus nerve in early PD patients(23).

Gut dysbiosis fosters α-synuclein misfolding through multiple convergent pathways. A state of chronic intestinal inflammation, driven by an imbalance in microbial communities and increased gut permeability, leads to the translocation of bacterial endotoxins such as lipopolysaccharide into the gut wall, triggering a local immune response characterized by elevated pro-inflammatory cytokines such as TNF-α and IL-1β. This inflammatory milieu within the enteric nervous system upregulates the expression of α-synuclein while simultaneously promoting its conversion into toxic, β-sheet-rich oligomers(24). Concurrently, dysbiosis-associated oxidative stress directly modifies the α-synuclein protein through nitration and carbonylation, rendering it more prone to aggregation. The inflammatory and oxidative insults also impair the cell's natural clearance mechanisms (eg, the ubiquitin-proteasome and autophagy-lysosome systems), allowing misfolded proteins to accumulate(25).

The mechanism of molecular mimicry is especially interesting. It is basically a situation where bacterial products directly contribute to human protein aggregation. For example, Escherichia coli produces functional amyloid fibers (curli), forming part of its biofilm. The curli subunit protein CsgA has the same structural motifs as human synuclein. These bacterial amyloids have the potential to act as external templates or seeds, and thus they can nucleate and speed up the aggregation of native synuclein through cross-seeding(26). Animal experiments show that colonization by curli-producing E. coli leads to an increase in synuclein pathology in both the gut and brain; thus, exposure over time to these bacterial amyloids may be the first step in disease development in genetically vulnerable subjects. In addition, the removal of beneficial microbial metabolites has a negative effect(27).

One of the PD hallmarks is the loss of SCFAs, especially butyrate. Butyrate is the main energy source for colonocytes and enteric neurons and has powerful anti-inflammatory and neuroprotective effects. With its reduction, the gut barrier becomes more permeable, there is a decrease in the expression of heat shock proteins that help in protein refolding, and the support from glial cells in the gut, which is necessary for the nourishment of neurons, is reduced; subsequently, this leads to a local disruption in protein homeostasis, which results in drastic synuclein misfolding and aggregation in enteric neurons. The gut pathology, once it has been set up, is ready to move upward(28).

Firstly, the vagus nerve, a key communication pathway that allows signals to travel in both directions between the brainstem and the abdomen, offers a direct anatomical connection. Pathological synuclein has prion-like features. It can be absorbed by neurons, and once inside, it can induce the misfolding of the normal protein in a cell-to-cell manner. The inflammatory state caused by dysbiosis could also promote this dissemination by elevating neuronal excitability and synaptic activity. The vital importance of the gut-brain axis is evidenced by epidemiological studies revealing that patients who had undergone truncal vagotomy showed a significantly reduced risk of PD even decades after the surgery(29). This gut-first model brings the microbial community back as a key, and potentially biochemically alterable, factor in PD development. It makes therapeutic intervention more targeted to the gastrointestinal tract at the earliest, likely prodromal phases of the disease. Such treatments include the use of specific prebiotics and probiotics, fecal microbiota transplantation, highly fermentable fiber dietary patterns, and therapies aimed at reconstruction of the intestinal barrier, all of which are capable of fundamentally modulating the disease course. These strategies, by alleviating the initial inflammatory and proteotoxic stimuli in the gut, are intended to block the chain reaction that eventually results in brain neurodegeneration, thus opening up a new and promising path(30).

## 3. Microbial Influence on Neuroinflammation: Oral Pathogen Focus

Recent research has broadened the perspective on neurodegenerative diseases, highlighting the role of microbial pathogens(31). Notably, Porphyromonas gingivalis, a periodontal pathogen associated with Alzheimer's disease, produces virulent proteases and can enter the CNS. This anaerobic bacterium evades immune responses, leading to long-lasting infections that can cause neurological damage. Its virulence factors, especially gingipains, are neurotoxic and stable across various pH levels, damaging proteins essential for neuronal health. In patients with Alzheimer's disease, gingipains are found near amyloid beta plaques and neurofibrillary tangles, suggesting that they directly contribute to disease mechanisms by cleaving amyloid precursor protein and disrupting protein degradation(32). Additionally, other microorganisms linked to neurodegenerative diseases include Treponema denticola, which produces dentilisin that may damage neural tissue, and Fusobacterium nucleatum, which is associated with colorectal cancer and Alzheimer's disease and promotes neuroinflammation. Even commensal species such as Streptococcus mutans can become neurotropic if they enter the bloodstream, suggesting that neurodegeneration may involve multiple pathogens rather than a single infectious cause(33).

Oral pathogens can reach the brain through various pathways, primarily hematogenous spread, whereby bacteria such as Porphyromonas gingivalis enter the bloodstream during dental procedures. It evades the immune response using mechanisms such as a capsule and the breakdown of complement factors. The bacteria can cross the BBB by migrating through cells or exploiting barrier disruptions. It can also travel via retrograde axonal transport through cranial nerves, such as the trigeminal nerve(34). Once in the CNS, Porphyromonas gingivalis causes persistent infections, evades full immune clearance, and survives within microglia and neurons, releasing virulence factors. This intracellular survival complicates antibiotic treatment and exposes neural tissue to bacteria, leading to neuroinflammation with altered cytokine levels and diminished phagocytic activity, which may exacerbate protein aggregate accumulation(35). Accordingly, periodontal pathogens contribute to systemic low-grade inflammation and impair the BBB, potentially affecting brain health. Inflammation can spread to the liver, where bacteria such as Porphyromonas gingivalis may contribute to metabolic disorders. Furthermore, gingipains from these bacteria can increase amyloid beta production and cleave tau proteins, promoting neurodegeneration. This process creates a cycle of neuroinflammation and cellular damage, exacerbating neurological decline(36,37).

Epidemiological studies suggest a connection between periodontal disease and cognitive decline, with chronic periodontitis linked to more rapid cognitive deterioration and increased dementia risk. Imaging studies reveal higher amyloid levels and brain shrinkage related to periodontal infections(38). Treatment with gingipain inhibitors reduces bacterial levels and improves symptoms associated with neurodegeneration. Additionally, female animals show more significant pathological changes, reflecting the heightened Alzheimer's disease risk in women(39). Recent discoveries have led to the development of new interventions for bacterial infections, focusing on small-molecule inhibitors that target gingipains, reduce bacterial virulence, and minimize the development of antibiotic resistance(40). Vaccines targeting Porphyromonas gingivalis surface antigens aim to prevent colonization, while adjuvant strategies promote better oral hygiene in high-risk groups and explore antimicrobial peptides with dual antibacterial and anti-inflammatory properties. Specifically, the goal is to disrupt the pathogenic processes without harming beneficial microbiota(41).

The gut microbiome influences the risk of neurodegeneration differently from oral bacteria. Pathogens such as Helicobacter pylori produce substances that can harm brain function through inflammation and nutritional deficiencies(42). For example, certain Escherichia coli strains may interact with human amyloid proteins, while excessive growth of Candida albicans can create neurotoxins. This highlights the importance of studying both oral and gut microbiomes in relation to neurodegenerative diseases(43). Moreover, the timing of microbial exposure is crucial for neurological outcomes, with chronic midlife infections causing cumulative damage that may lead to neurodegenerative disease(44). In particular, the prolonged prodromal phase of conditions such as Alzheimer's disease allows for complex interactions between microbes and risk factors, affecting individual vulnerability(45).

Genetic variations in immune response and host conditions, such as diabetes, influence susceptibility to neurodegeneration from infections such as periodontal disease(46). In addition, environmental and lifestyle factors that influence microbial risk can be modified to enhance prevention. Improved dental hygiene and regular check-ups may reduce harmful bacterial levels. A diet that supports a healthy oral and gut microbiome can help prevent the overgrowth of pathogens. Quitting smoking is vital because of its link to periodontal disease and weakened immunity(47). These steps offer straightforward methods to mitigate the risk of neurodegeneration in the population.

## 4. Mechanisms Linking Gut Dysbiosis to Neurodegeneration: Focus on Gut Barrier and Systemic Inflammation

Gut microbiota and neuroinflammation are two key intersecting points in understanding the pathogenesis of neurodegenerative diseases. New findings are bringing changes that occur in gut microbiota into focus as factors that can trigger, sustain, and modulate CNS functionality. This biophysical chain begins with the fundamental principle that the gut barrier is vulnerable to breakdown, and its integrity largely depends on controlling the microbes within(48). A healthy GI tract is selectively permeable, made possible by tightly coordinated epithelial junctions and mucus layers, which prevent the migration of luminal contents into the systemic circulation. This bowel wall is the initial line of defense that protects against microbial translocation; meanwhile, it still facilitates the absorption of nutrients(24). The gut microbiome plays a crucial role in maintaining this barrier function through various mechanisms, including the upregulation of mucus, regulation of tight junctions, and outcompetition of pathogenic microorganisms. Under circumstances in which microbial homeostasis is disrupted, due to antibiotic exposure, dietary alteration, chronic stress, etc, such delicate equilibrium is lost(49). Dysbiosis is a pathological imbalance of the resident microbiota, characterized by decreased niche stability, a decline in protective species, and an overgrowth of potentially pathogenic species within the host. This disproportion sets the stage for a “double hit” and drives enhanced gut mucosal permeability through several mechanisms(50). Some pathogenic bacteria secrete proteases and other enzymes that degrade tight junction proteins, including occludin and zonula occludens. Other microbes trigger the production of pro-inflammatory cytokines, which disrupt epithelial barrier function. The resultant disorder, now commonly referred to as leaky gut, allows bacterial products and metabolites to pass through the portal circulation and into systemic organs(51).

Lipopolysaccharides (LPS) are complex glycolipids that make up the outer membrane of Gram-negative bacteria. Normally, only small amounts of LPS can pass through the intestinal barrier and are efficiently eliminated by hepatic Kupffer cells(52,53). However, in conditions of dysbiosis and heightened permeability, substantial levels of LPS can enter the bloodstream, where they interact with pattern recognition receptors on immune cells. The LPS receptor complex, which includes CD14, MD2, and TLR4, triggers intracellular signaling pathways that activate nuclear factor kappa B and other transcription factors, leading to the production of inflammatory mediators. This systemic inflammatory response has profound implications for neurological health(54). Consistently, circulating pro-inflammatory cytokines, including tumor necrosis factor alpha (TNF-α), interleukin-1 (IL-1), and interleukin-6 (IL-6), have the potential to communicate with the CNS through several routes. These molecules may directly cross the BBB at circumventricular organs, where the barrier is naturally more permeable. Cytokines also bind to receptors on cerebral vascular endothelial cells, triggering secondary signaling within the brain parenchyma(55,56). Additionally, peripheral immune activation stimulates afferent vagal nerve fibers that project to brainstem nuclei and higher cortical regions involved in neuroimmune regulation(57). The BBB itself becomes both a target and mediator of neuroinflammation in this context. Chronic exposure to systemic inflammatory mediators alters the functional and structural integrity of the BBB through multiple mechanisms. Prolonged cytokine signaling leads to the downregulation of tight junction proteins between endothelial cells while increasing the expression of adhesion molecules that facilitate leukocyte trafficking. These changes create a positive feedback loop in which barrier dysfunction permits greater entry of inflammatory molecules into the CNS, further exacerbating neuroinflammation(58–61).

The mechanisms involved in microbial impact on neuroinflammation, including pathways beyond LPS-TLR4, are under investigation(62). Other factors, such as diet, stress, and physical activity, can modulate the gut microbiota, thereby affecting the risk of neuroinflammation. Persistently, comprehensive lifestyle interventions targeting gut health may offer neuroprotective strategies(63)(Table 1).

**Table 1:** Key Mechanisms Linking Gut Dysbiosis to Neurodegenerative Pathology

Key Mechanisms	Microbial trigger/mediator	Primary pathway	Neurodegenerative outcome
Protein aggregation	SCFA deficiency (↓ butyrate) Bacterial amyloids (curli from E. coli) Systemic inflammation (LPS, cytokines)	HDAC inhibition lost → ↑ BACE1 expression (18, 19) Molecular mimicry / cross-seeding (21, 26) Activation of GSK-3β, CDK5 kinases (19, 103)	Formation of Amyloid-β plaque; cognitive decline α-synuclein aggregation; Lewy body formation Tau hyperphosphorylation
Neuroinflammation	LPS translocation (leaky gut) SCFA deficiency (↓ butyrate, propionate)	TLR4 activation → NF-κB signaling (54, 88) Reduced HDAC inhibition; impaired GPR41/43 signaling (65, 89)	Neuroinflammation, neuronal loss, and microglial priming Pro-inflammatory microglial phenotype; impaired Aβ clearance
Barrier disruption	Dysbiosis-induced proteases Circulating cytokines (TNF-α, IL-1β)	Degradation of tight junction proteins (occludin, ZO-1)(24, 50) Downregulation of claudin-5, occludin (3, 58)	leaky gut and systemic inflammation BBB breakdown and CNS infiltration
Neurological dysfunction	LPS / Cytokines SCFA deficiency (↓ butyrate) Systemic inflammation	Microglial TLR4 activation (51, 88) Impaired astrocytic BDNF production; reduced glutamate uptake (EAAT2) (66, 90) ↑ TNF-α → oligodendrocyte apoptosis (92, 103)	Microglial priming Astrogliosis and impaired synaptic support Demyelination
Neurotransmitter Dysregulation	Dysbiosis-induced tryptophan metabolism shift Altered microbial production	↓ Serotonin, dopamine, and GABA synthesis (14, 70) ↑ iNOS, ↑ ROS/RNS production (89, 59)	Neuronal damage and cognitive impairment Impaired synaptic plasticity; mood dysregulation; motor symptoms
Oxidative Stress	LPS-induced inflammation Iron dysregulation	↑ iNOS, ↑ ROS/RNS production (58, 59) Hepcidin upregulation; altered iron metabolism (25, 93)	Impaired lipid peroxidation and mitochondrial function Ferroptosis susceptibility
Vagal Pathway	Gut-derived inflammatory signals α-synuclein propagation	Afferent vagal stimulation → nucleus tractus solitarius (57, 104) Retrograde vagal transport from ENS to CNS (23, 30)	Neuroinflammation Parkinson's disease

## 5. Gut-Brain Axis Biochemicals: Neuroactive Metabolites and Neurotransmitters

The gastrointestinal tract hosts a diverse microbial community that produces various neuroactive substances, creating a complex gut-brain interaction that affects physiological functions and cognitive abilities(64). Key metabolites include SCFAs, such as acetate, propionate, and butyrate, which are derived from the fermentation of undigested dietary fiber. Dietary habits influence SCFA levels, with plant-based, fiber-rich diets promoting their production, while Western diets typically result in lower levels. Butyrate, the most abundant SCFA, serves as an energy source for colonocytes, maintains intestinal barrier integrity, and exhibits neuroactive properties by crossing the BBB. It functions as a histone deacetylase inhibitor, altering gene expression in neurons and glial cells, thus affecting neuroplasticity, neurogenesis, and stress responses associated with neurodegeneration(65,66). Propionate, a 3-carbon fatty acid, has unique effects on the gut-brain axis, influencing gluconeogenesis in the liver and affecting metabolism. While normal levels support brain function, elevated levels have been linked to neurobehavioral problems in animals, possibly through modulation of neurotransmitters and the immune system. Acetate, the most abundant short-chain fatty acid, crosses the BBB and is vital for lipid synthesis and energy in brain cells. It also influences neuronal activity through G protein-coupled receptors, affecting excitability, synaptic plasticity, and neuroinflammation(67).

Gut microbiota influences neurotransmitter systems in addition to producing SCFAs. Serotonin, a key neurotransmitter that regulates mood, appetite, and cognition, is predominantly produced in the GI system, with gut microbes playing crucial roles(68). Some bacteria synthesize serotonin from tryptophan, while others influence its production by modifying the availability of the precursor or the activity of enterochromaffin cells. Additionally, gut microbes affect gamma-aminobutyric acid (GABA) metabolism, whereby various bacteria produce GABA from glutamate. This GABA can affect local physiology in the enteric nervous system and influence the host's overall responses, with links to stress and anxiety behaviors in animal studies(14). Dopamine, a crucial neurotransmitter associated with reward, motivation, and motor control, is influenced by the gut microbiota(69). Certain gut bacteria can convert dietary tyrosine into dopamine, while others produce metabolites affecting dopamine synthesis and receptor responsiveness. This interaction is significant for conditions such as Parkinson's disease, which is characterized by degeneration of dopaminergic neurons(70). Likewise, the gut microbiome can produce other neuroactive substances within a complex communication network between gut microbes and brain function, and dysbiosis disrupts this neurochemical balance, leading to neurodegenerative processes through diverse mechanisms such as compromised BBB integrity, reduced neurotrophic support, and increased neuroinflammation(71). Eventually, dysregulation of neurotransmitter production affects synaptic plasticity and neuronal viability in neurodegenerative disorders(72). Dysbiosis affects brain health by altering tryptophan metabolism, shifting it toward the kynurenine pathway, which increases excitotoxic neuroactive metabolites (eg, quinolinic acid) associated with neurodegeneration. Additionally, reduced butyrate production, as an SCFA, influences microglial function and neuroinflammation and contributes to neurodegenerative conditions(73).

The balance between neuroprotective and neurotoxic metabolites is crucial for brain health. In contrast, the gut microbiome's production of neurotoxic substances complicates the normal link between gut and brain in neurodegenerative diseases. Increased levels of bacterial metabolites such as D-lactic acid, ammonia, and phenolic compounds can be neurotoxic(74). Dietary modifications can influence microbial metabolite production, promoting the growth of beneficial bacteria that produce neuroactive compounds while reducing the growth of harmful ones. Resistant starches and polyphenol-rich foods support beneficial microbial communities and may lower the risk of neurodegenerative diseases(75,76).

Aging alters the gut microbiota, affecting metabolite profiles and increasing the risk of neurodegeneration in older individuals. Correspondingly, time-related patterns are crucial for developing strategies to boost microbial metabolite production(77). Differences in gut microbiota (eg, genetics, early life experiences, medication, and environment) lead to varied microbial metabolite production and influence the risk of neurological disorders. This highlights the potential for precision medicine targeting gut microbiome in neurological treatment(78,79). Additionally, it is essential to recognize that microbial metabolite profiles may serve as novel biomarkers for neurological disease risk, and strategies such as dietary adjustments and probiotics may aid in preventing neurodegeneration, but they necessitate careful consideration of the intricate relationships between diet, gut bacteria, metabolite production, and brain health(31).

## 6. Fundamental Pathways in Gut-Brain Communication

The enteric nervous system (ENS), comprising approximately 500 million neurons and often referred to as the second brain, is located in the gastrointestinal tract and maintains continuous communication with the vagus nerve, forming the gut-brain axis(80). Variations in vagal structure and function affect gut-brain axis responses, which are influenced by genetics and personal experiences that alter neural plasticity. These differences contribute to varying vulnerabilities to dysregulation and responses to treatments(81). The ENS has intrinsic neural circuits that process local information while also relaying selective signals to the CNS. Local circuits integrate signals from enteroendocrine cells sensing luminal contents and from interstitial cells of Cajal, which generate electrical activity in gut smooth muscle. The ENS features a variety of neurotransmitters that rival those of the CNS (eg, acetylcholine, dopamine, and serotonin), contributing to neuronal excitability and synaptic transmission(82). Gut signals travel through vagal afferents to the nucleus tractus solitarius, which relays this information to brain areas such as the hypothalamus and amygdala, thereby affecting mood and behavior. Descending vagal efferent fibers also connect with enteric neurons, allowing emotions to affect digestive functions. This bidirectional communication forms feedback loops between the gut and brain, highlighting their interdependence(83). In particular, acute stress significantly affects vagal function, activates the hypothalamic-pituitary-adrenal axis and sympathetic nervous system, and reduces digestive functions. Chronic stress leads to altered vagal afferent sensitivity, potentially causing gastrointestinal disorders and mood issues. In contrast, the gut microbiome influences vagal communication through mechanisms such as stimulation of the vagus nerve and effects on the enteric nervous system. For instance, SCFAs enhance vagal sensitivity to mechanical stimuli, while the microbiome maintains gut barrier integrity, thereby regulating vagus nerve activity by modulating immune-activating molecules(84).

Neuroinflammatory processes connect vagal signaling with neurodegeneration, where vagal afferents can relay pro-inflammatory signals to the brain, while vagal efferents counteract these effects through the cholinergic pathway. Disruptions in this neuroimmune interaction may hasten the progression of neurodegenerative diseases(85). For example, vagus nerve stimulation is being recognized as a potential treatment for gut-brain axis dysfunction associated with neurological disorders, demonstrating efficacy in conditions such as epilepsy, depression, and inflammation. These approaches seek to improve gut-brain communication by adjusting vagal tone and reducing inflammation, emphasizing crucial periods of adaptability within gut-brain pathways(86).

## 7. The Influence and Mechanisms of Gut Microbiome Disturbance on Neuroglial Elements

The gut microbiome is capable of influencing neuroglial cells in a very complex and profound way. Neuroglial cells include microglia, astrocytes, and oligodendrocytes, which are essential for CNS homeostasis, immune defense, and structural integrity. When there is a disturbance in gut microbiome composition (dysbiosis), fragile neuroglial homeostasis becomes disrupted, leading to neuroinflammation, synaptic dysfunction, and demyelination, which are typical features of neurodegenerative and neuropsychiatric diseases(87). Microglia are highly responsive to signals derived from the gut microbiome. Normally, microbial metabolites such as SCFAs, especially butyrate, play a major role in keeping microglia in a resting state in which they monitor their environment. By inhibiting histone deacetylases, butyrate shifts microglial gene expression toward an anti-inflammatory phenotype and also enhances their ability to move and phagocytose. Microglia in germ-free mice are immature both morphologically and functionally, and their ability to respond to injury is defective. This condition can be reversed either with SCFA treatment or by microbial recolonization. On the other hand, gut microbiome disturbance changes microglia to a state that is sensitized and overreactive(88). The leaky gut condition allows the passage of bacterial endotoxins such as LPS into the bloodstream. LPS interacts with microglial Toll-like receptor 4 (TLR4) and thus activates the NF-κB and MAPK pathways, resulting in the induction of pro-inflammatory cytokines (TNF, IL-1, IL-6) and reactive oxygen species generation. This chronic, low-grade inflammation leads to microglia becoming inefficient at clearing pathological protein aggregates (such as Aβ and synuclein) and synaptic debris. In addition, changes in tryptophan metabolism caused by dysbiosis shift precursors toward the kynurenine pathway, resulting in increased production of quinolinic acid, which stimulates microglial NMDA receptors and therefore aggravates excitotoxic neuroinflammation(89).

Astrocytes play a key role in synaptic maintenance, neurotransmitter recycling, and BBB integrity. Gut microbiota dysbiosis impairs astrocyte functionality through both direct and indirect effects. Circulating inflammatory cytokines and LPS induce astrocyte reactivity, leading to hypertrophy, increased expression of glial fibrillary acidic protein (GFAP), and alterations in neurotrophic factor secretion. Homeostatic features of reactive astrocytes, such as glutamate uptake via EAAT2, are commonly lost, thereby resulting in excitotoxicity. In addition, microbial metabolites affect astrocyte physiology. SCFAs, in particular propionate, regulate astrocytic metabolism and gene expression through G protein-coupled receptors (GPR41/43). Butyrate potentiates astrocytic production of brain-derived neurotrophic factor (BDNF), which supports neuronal survival and plasticity. Dysbiosis leads to a decrease in these advantageous metabolites, thereby resulting in astrocyte malfunction(90). Besides that, host-derived uremic toxins produced by gut microbiota (eg, indoxyl sulfate), in conditions such as chronic kidney disease, can cross the damaged BBB and cause oxidative stress in astrocytes. This further disrupts ion homeostasis and promotes neuroinflammation(91).

Oligodendrocytes myelinate axons, which helps rapidly transmit signals. Gut microbiota disturbance indirectly influences oligodendrocyte precursor cell (OPC) differentiation and mature oligodendrocyte survival. The systemic inflammation caused by dysbiosis is accompanied by an increase in proinflammatory cytokines that block OPC differentiation and lead to oligodendrocyte apoptosis. TNF, for example, stimulates death receptor pathways in oligodendrocytes, which is one of the reasons for demyelination in multiple sclerosis(92). SCFAs also affect oligodendrocyte physiology. Butyrate induces OPCs to differentiate into mature myelinating oligodendrocytes via epigenetic modulation of differentiation genes. A lack of SCFAs, such as in high-fat diets or antibiotic-induced dysbiosis, is associated with a decrease in myelination in animal models(92). Also, metabolites produced by the gut can regulate the pool of nutrients for lipid synthesis, which is very important in the production of myelin. Changes in bile acid metabolism caused by dysbiosis could also lead to changes in thyroid hormone signaling, which is considered a main regulator of oligodendrocyte development(93).

## 8. Therapeutic Implications of Gut-Brain Axis Modulation in Neurodegenerative Disorders

Emerging knowledge of the gut-brain axis in neurodegenerative diseases has led to the development of new therapeutic strategies aimed at influencing the microbiome-gut-brain axis, potentially modifying disease progression through microbial and metabolic adjustments. As nutrition significantly affects gut microbiota and is involved in brain health, dietary modification is a suitable therapeutic strategy(75). High-fiber diets promote the growth of beneficial bacteria and produce neuroprotective SCFAs. Additionally, various fibers, particularly resistant starches, enhance butyrate production and gut barrier integrity. Polyphenol-rich foods (eg, berries and dark chocolate) affect the gut-brain axis by enhancing beneficial microbes and producing neuroactive metabolites that can cross the BBB, which may help prevent neurodegenerative diseases. Probiotics, particularly Lactobacillus and Bifidobacterium strains, offer neurological benefits by modulating neuroinflammation and neurogenesis(94). However, the timing and diversity of fiber intake are crucial for maintaining microbial diversity and metabolic adaptability(95). Fecal microbiota transplantation involves transferring microbial communities from healthy donors to individuals with dysbiosis, originally for Clostridioides difficile infection but now also being considered for neurological applications to potentially restore gut-brain communication. Matching donors with recipients poses challenges, as optimal microbial compositions may differ for brain and gut health(96).

Strategies to reduce inflammation by improving gut permeability and preserving BBB integrity can effectively modulate microbes. Nutritional elements such as omega-3 fatty acids enhance tight junction proteins and stabilize membranes in intestinal and cerebrovascular endothelial cells(97). Phytochemicals such as curcumin offer anti-inflammatory and barrier-strengthening benefits, thereby alleviating systemic inflammation and its associated neurological effects(98). Lifestyle factors, including stress management through physical activity, significantly affect the gut-brain axis and are important for treatment strategies(99). Accordingly, personalized strategies for modulating the gut-brain axis are becoming more common, leveraging genetic, metabolic, and microbial information for tailored dietary advice and interventions, highlighting the variability in individual responses(100).

Medications originally designed for other purposes are being repurposed to affect the gut-brain axis, with certain diabetes treatments showing unexpected benefits in neurodegenerative models through mechanisms involving gut hormones and microbial metabolism. Some antidepressants also protect the gut barrier, potentially boosting their therapeutic effects(101). Advancements in methodologies for monitoring and assessing gut-brain axis interventions enable more accurate evaluations of therapeutic effects. For example, sophisticated sequencing provides insights into microbial community changes, while metabolic profiling assesses neuroactive metabolite production(102). Importantly, the safety considerations of gut-brain axis interventions require careful attention, particularly in patients with neurodegenerative conditions.

Abbreviations: SCFA, short-chain fatty acid; LPS, lipopolysaccharide; TLR4, Toll-like receptor 4; HDAC, histone deacetylase; BACE1, β-site amyloid precursor protein cleaving enzyme 1; GSK-3β, glycogen synthase kinase-3β; CDK5, cyclin-dependent kinase 5; BDNF, brain-derived neurotrophic factor; EAAT2, excitatory amino acid transporter 2; ENS, enteric nervous system; ROS, reactive oxygen species; RNS, reactive nitrogen species.

## Conclusion

The emerging body of evidence underscores the pivotal role of the gut-brain axis in the initiation and progression of neurodegenerative disorders. The gut microbiome, once viewed as a passive digestive component, is now recognized as an active modulator of neuroimmune, neuroendocrine, and neural signaling pathways that profoundly influence brain health. Gut dysbiosis, characterized by microbial imbalance and impaired gut barrier integrity, contributes to chronic systemic inflammation, increased permeability of both the intestinal and blood-brain barriers, and sustained microglial activation, key drivers of neuroinflammation and neuronal loss. In parallel, microbial alterations disrupt the production of neuroactive metabolites and neurotransmitters, which are essential for cognitive and emotional regulation. The involvement of pathogenic microorganisms, such as Porphyromonas gingivalis, further expands the landscape of neurodegeneration, suggesting that infectious agents may act as catalysts in the pathological cascade.

These mechanistic insights underscore the need to shift the therapeutic focus toward microbiome-centered interventions. Diet, probiotics, prebiotics, fecal microbiota transplantation, lifestyle modification, and anti-inflammatory bioactive molecules offer promising strategies to restore microbial equilibrium and protect neural function. However, significant gaps persist regarding individualized responses, optimal therapeutic timing, and long-term outcomes. Future studies must integrate microbial, genetic, and metabolic profiling to enable precision-based strategies for neurodegeneration prevention and therapy. Overall, strengthening gut-brain homeostasis presents an innovative and multidisciplinary approach that may transform our understanding and treatment of neurodegenerative diseases.

## Future Perspectives

Several key research areas need to be emphasized in future studies if we are to effectively translate current mechanistic knowledge into clinical applications. Comprehensive, large-scale, longitudinal cohort studies that deeply profile multi-omics aspects (metagenomics, metabolomics, proteomics) and combine these with high-resolution neuroimaging and clinical phenotyping are indispensable to delineate the time sequence of events, gut microbiota alterations, and the appearance of biomarkers and neuropathology during the prodromal stages of disease. These investigations will help determine whether microbiota dysbiosis is a primary driving force, an early reaction, or a secondary magnifying factor in neurodegenerative diseases. Equally, clinical translation hinges on the availability of dependable microbiota-based biomarkers. Microbial fingerprints, metabolite signatures, or their combinations might facilitate not only early diagnosis but also tracking disease progression and anticipating therapeutic efficacy. Nevertheless, the quest for potent biomarkers can only be successful if there is standardization of methodological approaches and validation in a variety of populations(105,106).

There are several difficulties in designing such clinical trials that must be considered. Firstly, the initial composition of microbes varies greatly from one person to another; a uniform intervention might not work for everyone. Secondly, it is hard to devise a placebo for those who have undergone dietary or lifestyle changes. Thirdly, a typical microbiome-targeted intervention might also affect other parts of the microbial community unintentionally. Lastly, there is no agreement as to what should be measured when assessing the outcome. Future research should consider creative approaches such as adaptive trial design and selection of participants based on their microbial signatures. Also, using markers of the mechanism of action that show how therapies work would be beneficial(79).

One step further in finding out the causal relationships is the transition from simply discovering microbial signatures to the deconstruction of specific host-microbe molecular interactions at the level of individual molecules. One way to achieve this is through experiments with humanized gnotobiotic animal models colonized with defined microbial consortia from patients, combined with single-cell RNA sequencing of neuroglial cells and brain spatial metabolomics. A key focus should be on uncovering the identities of particular bacterial strains, enzymes, and metabolites that strongly modulate microglial phagocytosis, astrocytic reactivity, and neuronal resistance to ferroptosis or excitotoxicity(107). Due to the microbiome's extremely unique nature in each individual, this area of research inevitably has to shift toward personalized medicine approaches. Future research needs to be redirected toward creating predictive models that combine a person's microbial profile, immune status, genetic susceptibility, and lifestyle factors to predict the course of disease and the response to treatments targeting the microbiome. This model would make it possible to design personalized interventions on a rational basis that may include custom prebiotic and probiotic formulations, next-generation “neurobiotics,” or fecal microbiota transplantation from the best donor match(108).

## Data Availability

No new data were generated..
